# Individual altruistic choice and attitude data from Amazon׳s Mechanical Turk

**DOI:** 10.1016/j.dib.2018.07.052

**Published:** 2018-07-27

**Authors:** Vera L. te Velde

**Affiliations:** University of Queensland, Australia

## Abstract

This article presents new data on individual altruistic choices and beliefs in a two-player sharing game, collected for the study reported in te Velde (2018) [2]. The decision maker in this game had to choose how much of a fixed budget to share with another anonymous Amazon Mechanical Turk user participating in the same study. With a known probability, the decision maker׳s choice was overridden by one of two known default options, so that the recipient was unable to attribute those two outcomes to the decision maker with certainty. In addition to choice data, each participant׳s incentivized guess of average behavior is included in the dataset, along with responses to a set of survey questions asking for opinions or beliefs about the morality of different options or the preferences or experienced utility of the recipient. Lastly, the dataset contains basic demographic information for each participant, along with detailed timing information about recruitment and each phase of the study.

**Specifications Table**

**Table:** 

Subject area	*Economics, psychology*
More specific subject area	*Behavioral economics, experimental economics, social psychology*
Type of data	*Text file*
How data was acquired	*Human participants in an incentivized game and survey administered through Amazon׳s Mechanical Turk*
Data format	*Raw*
Experimental factors	*N/A*
Experimental features	*Individual choices and beliefs in a sharing game*
Data source location	*N/A*
Data accessibility	*Data is included in the online supplementary material for this article*
Related research article	*te Velde (in press)*[2]*; te Velde (2018)*[3]

**Value of the data**

• These data are useful for studying how people make altruistic choices.
• Rather than simple revealed-preference data, the dataset reveals aspects of the decision makers’ thought processes, such as their expectations of others’ behavior in the same setting and their beliefs about the recipient׳s preferences and emotional reactions to potential outcomes.
• Timing and demographic data included in the dataset is of value for examining how different types of decision makers proceed through each phase of the game.
• The dataset contains observations of a large number of decision makers, allowing researchers to break down the data however desired without losing statistical power.

## 1 Data

The data contains observations from an incentivized economic experiment and survey implemented on Amazon׳s Mechanical Turk (MTurk) platform with 2306 participants. The study consisted of three phases: a game, a questionnaire, and a survey. The game was played either before or after the questionnaire depending on the treatment group, and the survey came last. The game is an adapted version of the modified dictator game first implemented by Andreoni and Bernheim [1]. The dictator chooses how much of $1.00 to share with a recipient, and with a commonly known probability this choice is overridden with one of two default allocations. The survey collected basic demographic data on gender, race, income, education, and age.

The questionnaire is the primary innovation provided by this dataset. It asks the dictator a set of eight novel questions eliciting beliefs about others’ beliefs, experienced utility, and preferences. Specifically, each participant is asked to state their opinions regarding the active choice of the default allocations, which cannot be directly attributed to the dictator, versus choices very close in monetary value to one of the default options. Depending on the treatment arm, dictators are asked either about the relative moral appropriateness of these options, or which option they believe the recipient would prefer for the dictator to choose, or which option they believe would lead the recipient to be happier.

## 2 Experimental design, materials, and methods

### 2.1 Recruitment

Participants were recruited with a posting on MTurk advertizing a baseline payment of $0.40. Any MTurk user located in the United States was able to participate; I additionally discarded responses from IP addresses located outside the United States or multiple responses from the same IP address. Upon accepting the task, participants were assigned to a treatment group by assigning sequential participants to sequential treatments. They were directed to a private web server hosting the experiment, which also recorded timing information as they progressed through each phase of the study. After all participants had completed the study, they were anonymously paired in order by arrival time and one partner׳s choice was enacted with 20% probability. Final payments were made with bonuses through MTurk, accompanied with an explanation of all results. All aspects of the setting were commonly known.

Demographic information of participants in summarized in Table 1 of te Velde [2].

### 2.2 Procedures

After agreeing to the terms laid out in the consent form, the study consisted of three phases; the game, questionnaire, and survey. Each will be described in turn:

*Game:* The game is adapted from the design of Andreoni and Bernheim [1]. Example instructions are included in Appendix A in the online supplementary material. This is two-player modified dictator game in which the dictator chooses how much of $1 to share with the recipient. This choice is recorded in the “shared” field in the dataset. Then with a commonly known probability, the dictator׳s choice is overridden with one of two commonly known default allocations (each with equal probability). One of these possible allocations gives the dictator 9 cents and the remaining 91 to the recipient, and in the other these two payments are reversed. The resulting allocation is communicated to the recipient, but not the dictator׳s original choice or whether it was overridden.

Prior to making an allocation decision, participants also guessed the median allocation decision among other participants within their treatment group. This guess was incentivized for accuracy by paying 5 cents if the true median was within 10 cents of the guess. It is recorded in the “sharedguess” field in the dataset.

*Questionnaire:* An example questionnaire is included in Appendix B in the online supplementary material. The questionnaire presents the same setting as the game, but refers to hypothetical dictator Alice and recipient Bob. Additionally, the total amount Alice can share with Bob is $10 and the two possible default allocations give 87 cents to Alice and $9.13 to Bob, or vice versa. The first question simply asks people to state their opinion of how much they think Alice would share with Bob, and the second question is an incentivized guess of the median response to the first question within the participant׳s treatment group. If the true median was within 25 cents of the participant׳s guess, they were paid an additional 5 cents. These two responses are recorded in the “guess” and “metaguess” fields in the dataset, respectively.

The main questionnaire consisted of 8 questions. Each question compares two allocations, one of which is one of the two default allocations, d, and the other is slightly larger or smaller than that default allocation, d±ϵ. For example, the first two questions in the example questionnaire in Appendix B uses d=87 cents and ϵ=5, so the participant must first compare 87 cents to 92 cents and then 87 cents to 82 cents. These are very similar in monetary outcome, but the latter comes with definitive information that Alice was actively ungenerous. A response favoring 87 over 92 cents therefore communicates that bad information is worth at least 5 cents to avoid, and is recorded with a 1 in the “badinfobad” field in the dataset. Similarly, favoring 82 cents over 87 cents indicates that bad information is worth at least 5 cents, and such a response is recorded with a 1 in the “badinfogood” field. “goodinfobad” and “goodinfogood” are defined analogously.

Questions 7–10 are the same as questions 3–6, but use a larger value of ϵ. These correspondingly detect stronger preferences over good and bad information. For each of questions 3–10, the order of options was randomized for each participant. Also, in the dataset, responses to the last four questions are missing for 1439 subjects due to a glitch in the experiment software that was rectified partway through the study.

*Survey:* The survey is included in Appendix C of the online supplementary material. These responses are recorded in the fields “agebracket”, “gender”, “ethnicity”, “income”, and “educ”.

### 2.3 Treatments groups

Each participant was assigned to one of 24 treatments, using a 2 × 2 × 2 × 3 design. Treatment assignment is recorded with a four-digit code in the “treatment” field of the dataset. Each digit, respectively, designates the following treatment dimensions:

1. “Game first” versus “game second”. The former is designated with a 2 in the first digit and indicates that participants played the game prior to completing the questionnaire, (both described in the next subsection). All participants also completed a survey after everything else.
2. “Small values” versus “large values”. The former is designated with a 1 in the second digit and indicates that the questionnaire used values of ϵ=5 and 40 cents. A 2 in the second digit indicates that the questionnaire used values of ϵ=20 and 70 cents.
3. “High school pressure” versus “low social pressure”. The former is indicated with a 2 in the third digit and specifies that the probability of the dictator׳s choice being overridden by the computer was 10%, versus 50% in the low pressure treatment, both in the game and in hypothetical game considered in the questionnaire.
4. Question phrasing: In the “ex post happiness” versus “ex ante preference” versus “moral appropriateness”, designated respectively with a 1, 2, or 3 in the fourth digit. The “ex post happiness” treatment phrased the questionnaire questions as “Do you think Bob would be happier after receiving a payment of” either of two possibilities. In the “ex ante preference” treatment, participants were instead asked “Do you think Bob would prefer for Alice to choose to share” either of two possibilities. In the “moral appropriateness” treatment participants were asked “Do you think it׳s more morally appropriate for Alice to choose to share” either of two possibilities.

### 2.4 Ethical considerations

This study was approved by the University of California, Berkeley Committee for the Protection of Human Subjects. All participants read and actively consented to the terms of the study, which maintained anonymity of participants and permitted them to quit at any time without penalty. Participants were informed prior to accepting the MTurk task that their payments would depend on their own actions, others’ actions, and on chance. The specific goals of the study were not explained in order to minimize experimenter demand effects; however participants were told that the MTurk task was a study on economic decision making.

### 2.5 Applications to behavioral economics and social psychology

Game data is essentially an online replication of the Andreoni and Bernheim [1] experiment. This data therefore is useful as a comparison of social pressure and altruism in the lab versus online and with smaller stakes, which is relevant to both the science of these phenomena and to methodological considerations when designing lab or online experiments.

The questionnaire data is entire novel to this dataset. This data is useful for researchers interested in the non-instrumental utility of beliefs and the morality of manipulating beliefs directly in cases in which ignorance may be bliss or when information comes at a price, both instrumental and emotional. This may therefore be of interest to social or cognitive psychologists in addition to other behavioral economists.

The demographic fields from the survey were collected as control variables and were not part of the randomized experimental design; however, suggestive evidence on demographic comparisons of social preferences and beliefs that this dataset enables may be useful for other researchers when designing further studies on these issues.
