# Supplementary material for: Individual altruistic choice and attitude data from Amazon׳s Mechanical Turk
Source: Data Brief. 2018 Jul 27;20:210–4. doi: 10.1016/j.dib.2018.07.052 (PMC6098210; doi:10.1016/j.dib.2018.07.052)
Supplement: Supplementary file 1 — Transparency document [file mmc1.pdf]

THE UNIVERSITY OF QUEENSLAND
AUSTRALIA

Vera te Velde
University of Queensland School of Economics
Colin Clark Bldg. 39 Level 6
St. Lucia, QLD, 4072
Australia

I, the author of the data paper “Individual altruistic choice and attitude data from Amazon’s Mechanical Turk”, declare that there are no conflicts of interest relevant to the publication of this article.

Signed,

Vera te Velde

Vera te Velde
