# Supplementary material for: Individual altruistic choice and attitude data from Amazon׳s Mechanical Turk
Source: Data Brief. 2018 Jul 27;20:210–4. doi: 10.1016/j.dib.2018.07.052 (PMC6098210; doi:10.1016/j.dib.2018.07.052)
Supplement: Supplementary file 3 — Supplementary material [file mmc3.pdf]

Appendix A: Example game instructions

Welcome!

My name is Vera te Velde, and I am a researcher at the University of California at Berkeley. This short set of questions will help us understand how you think about certain economic and social situations.

There are no right or wrong answers, but you must answer carefully and honestly in order to avoid directly contradicting yourself (for example, by saying you prefer an apple to an orange, but you'd rather have an orange than *two* apples). As long as you answer honestly, you don't need to worry about making that kind of mistake, and you will be eligible for bonus payments!

Game

First, you will have the opportunity to play a simple game for real money with a real partner. You have been matched with another MTurk user. You will both play the exact same game, shown below. Either your choice or your partner's choice will be randomly selected, and then with a 20% probability, the game will count for real money and you will be paid according to the results with an MTurk bonus payment. The results of the game will be reported to you on MTurk after both you and your partner have completed the HIT, along with your bonus payment (if applicable).

In this game, you must decide how to divide $1 between yourself and your partner. After you decide, the computer will draw a random number, and half the time it will override your choice. If it does, half the time it will give 9 cents to you and 91 cents to your partner, and half the time it will choose to give 91 cents to you and 9 cents to your partner. Your partner will see the outcome of the game, but will *not* find out whether the computer overrode your choice.

Overall, 25% of the time the computer will share 9 cents with your partner, 25% of the time it will share 91 cents, and half the time it will share whatever amount you choose below.

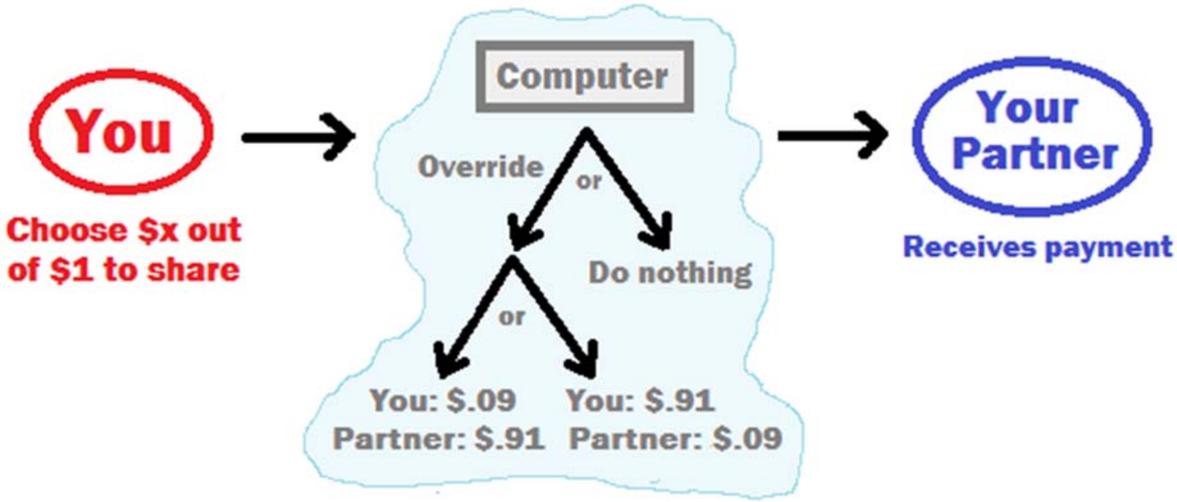

1. How much would you like to share with your partner? Choose an amount between $0 and $1: $1 ▼

2. What do you think most other people will answer to the previous question? For this question, if you guess within 10 cents of the middle answer among MTurk users who complete this HIT, you will receive a 5 cent bonus payment! $1 ▼

If you have any thoughts or comments, or if you would like to share your reasoning, please do so here. We really appreciate your input!

Continue

This is a study by the University of California, Berkeley, Department of Economics. For questions, contact Vera te Velde at vtevelde@econ.berkeley.edu.

Appendix B Example questionnaire instructions

Questionnaire

Next I will ask you a few questions about a game very similar to the one you just played. Imagine the following scenario. Two people, Alice and Bob, are playing a game through a computer. They don't know each other and will never actually meet, and they are playing this game together just one time.

In this game, Alice must decide how to divide $10 between herself and Bob. After she decides, half the time they each receive their money and the game ends. The other half of the time, the computer randomly decides to override Alice's choice. Half the time the computer decides to give Alice $9.13 and Bob $0.87, and half the time it gives Alice $0.87 and Bob $9.13.

Alice and Bob know the rules of the game, and Bob can see how much money he receives, *but he doesn't know whether the computer actually intervenes or what it randomly chooses*.

Overall, 25% of the time the computer shares $0.87 with Bob, 25% of the time it shares $9.13 with Bob, and half the time it shares whatever quantity Alice chooses.

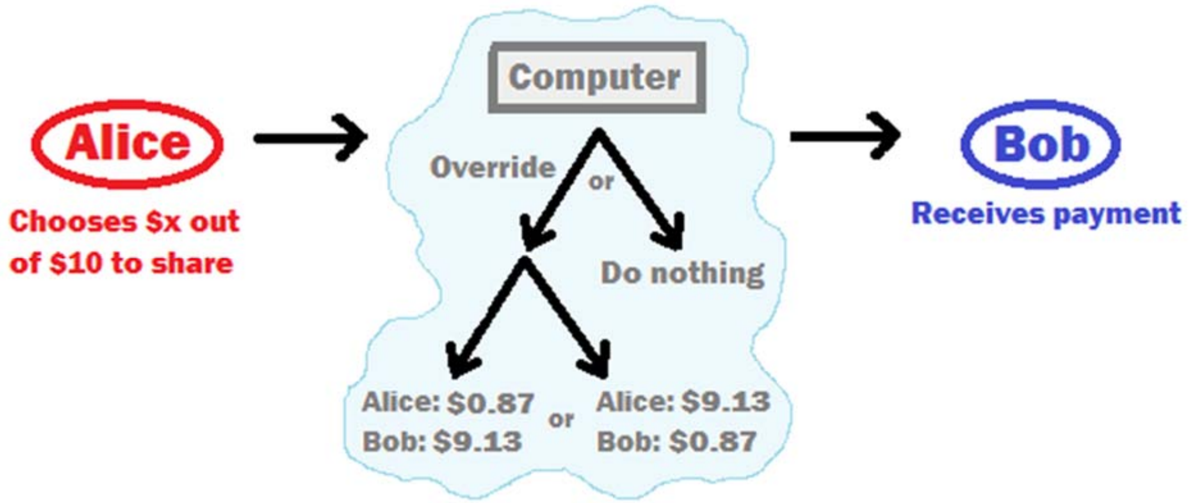

Notice: If Alice chooses to give Bob either $0.87 or $9.13, Bob cannot figure out whether Alice actually chose to share that amount or whether the computer overrode her choice. If Alice chooses to give Bob any other amount, he can figure out what she chose.

The following few questions ask you to guess or express an opinion about some of the possible outcomes in this game. Imagine that Bob and Alice are average people and answer what you think they would do or think.

1. How much do you think Alice will choose to share with Bob? Enter an amount between $0.00 and $10.00.

2. What do you think most other people will answer to the previous question? For this question, if you guess within 25 cents of the middle answer among other MTurk users who complete this HIT, you will receive a 5 cent bonus payment!

3. Do you think Bob would be happier after receiving a payment of $0.87 or $0.92?
   - $0.87
   - $0.92

4. Do you think Bob would be happier after receiving a payment of $0.87 or $0.82?
   - $0.87
   - $0.82

5. Do you think Bob would be happier after receiving a payment of $9.13 or $9.18?
   - $9.13
   - $9.18

6. Do you think Bob would be happier after receiving a payment of $9.13 or $9.08?
   - $9.13
   - $9.08

7. Do you think Bob would be happier after receiving a payment of $0.87 or $1.27?
   - $0.87
   - $1.27

8. Do you think Bob would be happier after receiving a payment of $0.87 or $0.47?
   - $0.87
   - $0.47

9. Do you think Bob would be happier after receiving a payment of $9.13 or $9.53?

- $9.13
- $9.53

10. Do you think Bob would be happier after receiving a payment of $9.13 or $8.73?

- $9.13
- $8.73

If you have any thoughts or comments, or if you would like to share your reasoning, please do so here. We really appreciate your input!

Next

This is a study by the University of California, Berkeley, Department of Economics. For questions, contact Vera te Velde at vtevelde@econ.berkeley.edu.

Appendix C Survey

Survey

Please answer the following five questions to complete the study:

What is your age bracket?

- Under 30
- 30-39
- 40-49
- 50-59
- 60 or over
- I prefer not to answer

Are you male or female?

- Female
- Male
- Other, or I prefer not to answer

What is your ethnicity?

- White
- Non-white Hispanic
- Black or African American
- Asian or Pacific Islander
- Other
- I prefer not to answer

What is your household income?

- Less than $20,000
- $20,000 to $49,999
- $50,000 to $79,999
- $80,000 to $149,999
- $150,000 or more
- I prefer not to answer

What is your highest completed education level?

- High school or less

- Some college
- Bachelor’s degree
- Postgraduate degree
- I prefer not to answer

Finish

This is a study by the University of California, Berkeley, Department of Economics. For questions, contact Vera te Velde at vtevelde@econ.berkeley.edu.
